# Dietary steamed wheat bran increases postprandial fat oxidation in association with a reduced blood glucose-dependent insulinotropic polypeptide response in mice

**DOI:** 10.1080/16546628.2017.1361778

**Published:** 2017-08-23

**Authors:** Yayoi Hosoda, Fumiaki Okahara, Takuya Mori, Jun Deguchi, Noriyasu Ota, Noriko Osaki, Akira Shimotoyodome

**Affiliations:** ^a^ Biological Science Laboratories, Kao Corporation, Tochigi, Japan

**Keywords:** Arabinoxylan, glucose-dependent insulinotropic polypeptide, fat oxidation, obesity, wheat bran

## Abstract

Obesity is a global epidemic associated with a higher risk of cardiovascular disease and metabolic disorders, such as type 2 diabetes. Previous studies demonstrated that chronic feeding of steamed wheat bran (WB) decreases obesity. To clarify the underlying mechanism and the responsible component for the anti-obesity effects of steamed WB, we investigated the effects of dietary steamed WB and arabinoxylan on postprandial energy metabolism and blood variables. Overnight-fasted male C57BL/6J mice were fed an isocaloric diet with or without steamed WB (30%). Energy metabolism was evaluated using an indirect calorimeter, and plasma glucose, insulin, and glucose-dependent insulinotropic polypeptide (GIP) levels were measured for 120 min after feeding. We similarly investigated the effect of arabinoxylan, a major component of steamed WB. Mice fed the WB diet had higher postprandial fat oxidation and a lower blood GIP response compared with mice fed the control diet. Mice fed the arabinoxylan diet exhibited a dose-dependent postprandial blood GIP response; increasing the arabinoxylan content in the diet led to a lower postprandial blood GIP response. The arabinoxylan-fed mice also had higher fat oxidation and energy expenditure compared with the control mice. In conclusion, the findings of the present study revealed that dietary steamed WB increases fat oxidation in mice. Increased fat oxidation may have a significant role in the anti-obesity effects of steamed WB. The postprandial effects of steamed WB are due to arabinoxylan, a major component of WB. The reduction of the postprandial blood GIP response may be responsible for the increase in postprandial fat utilization after feeding on a diet containing steamed WB and arabinoxylan.

## Introduction

The number of obese people worldwide is increasing as a result of sedentary lifestyles and the spread of a Western-style diet. Obesity is a risk factor for diabetes, dyslipidemia, hypertension, arteriosclerosis, and various cancers. These obesity-related disorders critically decrease a patient’s quality of life. Therefore, prevention and improvement of obesity are important health goals worldwide. Many studies report that dietary macronutrient distribution patterns, such as those supported by the Ornish [1] and Atkins diets [2], and specific nutritional components, such as dietary fiber (DF), are effective for the primary prevention and treatment of these diseases [3].

A number of epidemiological and intervention studies revealed that whole grains or various grain brans have protective effects against obesity, metabolic syndrome, diabetes mellitus, cardiovascular disease, and cancer [4–9]. Wheat bran (WB) is the outer covering of the wheat grain, and contains abundant vitamins, minerals, and DF [10]. Chronic consumption of WB effectively improves obesity in mice [11,12]. Han et al. [11] demonstrated that chronic feeding with WB induces the expression of proteins involved in fat oxidation and suppresses the expression of proteins involved in fatty acid synthesis in liver and epididymal adipose tissue. Moreover, chronic feeding of WB induces lipolysis and browning of white adipose tissue in mice [12]. Harding et al. [13] reported that autoclaved WB has anti-obesity effects in rodents. Furthermore, several studies indicate that short-term supplementation with WB has beneficial effects, such as nutrient excretion into the stool [14], glucose-lowering effects [15], and decreased gastric emptying effects [16,17]. Little is known, however, about the effect of a single ingestion of WB on energy metabolism.

Glucose-dependent insulinotropic polypeptide (GIP) is a gastrointestinal incretin hormone that stimulates the secretion of insulin from pancreatic βcells. GIP is secreted from K cells located in the proximal region of the small intestine (duodenum and jejunum) through the activity of secretagogues, such as dietary fat and carbohydrates. Although the actions of GIP to promote insulin secretion from the pancreatic βcells are widely known, extrapancreatic actions of GIP/GIP receptor (GIPR) signaling in relation to obese phenotypes are also well described [18–25]. GIP has various anabolic effects via GIPRs on adipocytes, including stimulation of glucose uptake, lipoprotein lipase activity [18,19], and fatty acid synthesis [20]. Genetically GIPR-ablated mice exhibit high fat utilization and resistance to high-fat induced obesity [21,22]. In addition, daily injection of a specific GIPR antagonist protects mice against obesity [23,24]. Together, these lines of evidence indicate that inhibiting GIP signaling can prevent obesity. On the other hand, many studies indicate that not only inhibition of GIPR, but also control of GIP secretion is effective for preventing obesity. A genetic deficit in GIP-secreting K cells enhances energy expenditure and prevents high-fat diet-induced obesity in mice [25]. In contrast, intravenous administration of GIP to healthy lean men lowers resting energy expenditure (REE) [26]. In our previous study, we found that the dietary components that control GIP secretion, such as diacylglycerol [27], 1-monoolein [28], and RS4-type-resistant starch [29], increase postprandial fat utilization and prevent high-fat diet-induced obesity in mice. Reduction of postprandial GIP secretion can increase postprandial energy catabolism and prevent obesity.

Accordingly, the aim of the present study was to clarify the underlying mechanism and the responsible component for the anti-obesity effect of steamed WB. We investigated the postprandial effects of dietary steamed WB and arabinoxylan, a major component of WB, on postprandial energy metabolism and the blood response of anabolic hormones, such as GIP and insulin, in mice.

## Materials and methods

### Animals and dietary materials

Male C57BL/6J mice (9 weeks old) were obtained from Clea Japan (Tokyo, Japan), and maintained at 23 ± 2°C under a 12:12 h light–dark cycle (lights on from 7.00am to 7.00pm). The mice were individually housed in plastic cages and fed a laboratory diet (CE-2; Clea Japan) for 1 week to acclimatize them to the metabolic conditions.

WB was purchased from Nisshin Pharma (Wheat bran DF; Tokyo, Japan). The WB was steamed using a twin screw extruder (EA-20; Suehiro EPM Corp., Mie, Japan) and termed ‘steamed WB’. The nutritional content of the steamed WB is shown in Table 1. Milk casein, corn oil, cellulose, AIN-76 mineral mixture, AIN-76 vitamin mixture, and gelatinized potato starch were purchased from Oriental Yeast Co. (Tokyo, Japan), and sucrose was purchased from Wako Pure Chemical Industries (Osaka, Japan). Lignin was purchased from Kanto Chemical Co. (Tokyo, Japan).

**Table 1.** Nutritional content of steamed wheat bran.

Component	Content (%)
Protein	18.9
Fat	4.2
Carbohydrate	20.2
Dietary fiber	47.9
Moisture	2.3
Ash	6.5

### Preparation and characterization of arabinoxylan fraction from steamed WB

The arabinoxylan fraction was prepared according to previously reported methods [30–32]. In brief, the steamed WB was washed with hexane, 99.5% ethanol, and distilled water, and then heated in 0.4 M NaOH aq. at 80°C for 1.5 h and filtered. The filtrate was neutralized with 2.0 M HCl aq. and incubated with α-amylase, protease, and amyloglucosidase (Sigma-Aldrich, St Louis, MO, USA). The mixture was filtered and then adjusted to 65% (v/v) ethanol and allowed to stand at 4°C for 1 h. The resulting precipitate was collected after centrifugation at 3000 *g* for 15 min at 5°C, washed twice with 70% (v/v) ethanol, ground in 99.5% ethanol, and washed with acetone. The precipitate was then dried to obtain the carbohydrate isolates as the arabinoxylan fraction. It was found that 60 g of steamed WB produced approximately 12 g of arabinoxylan fraction.

The arabinoxylan content was determined as the total amount of arabinose and xylose after hydrolysis of the fraction using a high-performance liquid chromatography system with a refractive index detector (HPLC-RI detector, Elite Lachrom; Hitachi, Tokyo, Japan) and high-performance anion exchange chromatography (DX-500; DIONEX, Sunnyvale, CA, USA). The protein content was calculated using 6.25 as the nitrogen to protein conversion factor to convert nitrogen content (%) to protein (%). Nitrogen was measured using a SUMIGRAPH NCH-22F elemental microanalyzer (Sumika Chemical Analysis Service, Osaka, Japan). Molecular weight was determined by a size-exclusion HPLC-RI detector using molecular standards of pullulan. Phytic acid was determined by anion-exchange column chromatography using a conductivity detector. The composition of the arabinoxylan fraction is shown in Table 2.

**Table 2.** Composition of the arabinoxylan fraction.

Component	Content (%)
Arabinose/xylose	58.5
Phytic acid	19.4
Protein	7.1
Other	15.0

### Experimental diets and animal studies

We placed a dome-type cover on the feeding dish (Roden CAFE; Oriental Yeast Co.) to avoid scattering of the powdered diet in the cage. The mice were first fed a low-fat powder diet (Table 3) for 3 days to acclimatize them to the powder diet before each experiment.

**Table 3.** Nutrient contents of diets used in Experiments 1 and 2.

	Nutrient content (mg/g diet)		
Ingredient	Low-fat diet	Control diet	WB diet
Gelatinized potato starch	665	419	286
Sucrose	0	130	130
Corn oil	50	81	69
Milk casein	200	183	126
Cellulose	40	142	44
AIN-76 mineral mixture	35	35	35
AIN-76 vitamin mixture	10	10	10
Steamed WB	0	0	300
Arabinoxylan	0	0	89.4
Total energy (kJ/g)	16.4	15.3	15.3
Fat (% energy)	11.5	60.1	60.1
Protein (% energy)	20.5	20	20
Carbohydrate (% energy)	68.0	19.9	19.9

WB, wheat bran.

In Experiment 1, the mice were habituated to a metabolic cage for 2 days of respiratory analysis, and then divided into two groups (*n* = 8 per group): control and steamed WB groups. The means and standard deviations (SDs) of body weight (BW) were counterbalanced between groups. Mice in the control group were fed a control diet (Control diet), and mice in the WB group were fed a diet containing 30% steamed WB (WB diet). The nutrient contents of the diets are shown in Table 3. To equalize the energy content of each diet, isocaloric protein, lipid, and carbohydrate were added to the Control diet but not the steamed WB. After overnight fasting, the mice were fed the experimental diets (3.83 kJ) and postprandial respiratory metabolic performance was measured by an indirect calorimetric system equipped with a 16-chamber airtight metabolic cage (Arco2000; Arco System, Chiba, Japan). Airflow through the metabolic cage was adjusted to 0.32 L/min. Data were collected continuously at 15 s intervals for a total of 120 min, and the reference was room air. Data were obtained every 5 min for each chamber. The substrate utilization and REE were calculated from the measured values of oxygen consumption (*V*O_2_) and carbon dioxide production (*V*CO_2_) according to our previous study [33]: carbohydrate oxidation (mg/g BW/min) = 4.113 × *V*CO_2_ – 2.907 × *V*O_2_, fat oxidation (mg/g BW/min) = 1.689 × (*V*O_2_ – *V*CO_2_), and REE (J/g BW/min) = 16.38 × *V*O_2_ + 4.62 × *V*CO_2_. Locomotor activity was measured using an automated motion-detection system (Actracer-2000; Arco System) that detects the amount of centroid fluctuation using a weighted transducer. Total amounts of fat, carbohydrate, and energy consumed, and physical activity were measured for 120 min after providing the food to the mice.

In Experiment 2, overnight-fasted mice were divided into two groups (*n* = 16 per group): control and WB groups. The means and SDs of BW and the fasting blood glucose concentration were adjusted between groups. Mice were fed equal amounts (3.83 kJ) of the diets for 30 min. Using a heparinized capillary tube (Kimble Chase, Rochester, NY, USA), blood samples were collected from the orbital sinus under anesthesia with 2.5% isoflurane inhalation at 0, 30, 60, and 120 min after providing the experimental diet. The blood samples were kept on ice until plasma preparation. After centrifugation at 10,000 *g* for 6 min at 4°C (MIKRO 22R, Hettich), the plasma was stored at –80°C until analysis.

In Experiment 3, overnight-fasted mice were divided into three groups (*n* = 8 per group): dietary fiber-free (DF-free), low-dose arabinoxylan (L-AX), and high-dose arabinoxylan (H-AX) groups. The means and SDs of BW and fasting blood glucose concentrations were counterbalanced among groups. Mice in the DF-free, L-AX, and H-AX groups were fed diets containing 0%, 5.3%, or 9.8% arabinoxylan, respectively (Table 4). As described in Experiment 2, the experimental diets were fed to the mice, and postprandial blood samples were collected.

**Table 4.** Nutrient contents of diets used in Experiments 3 and 4.

	Nutrient content (mg/g diet)		
Ingredient	DF-free diet	L-AX diet	H-AX diet
Gelatinized potato starch	510	464	425
Sucrose	130	118	108
Corn oil	97	88	81
Milk casein	218	198	182
AIN-76 mineral mixture	35	32	29
AIN-76 vitamin mixture	10	9	8
Arabinoxylan fraction	0	91	167
Arabinoxylan	0	53.2	97.6
Total energy (kJ/g)	18.0	16.4	15.0
Fat (% energy)	20.3	20.3	20.3
Protein (% energy)	20.3	20.3	20.3
Carbohydrate (% energy)	59.4	59.4	59.4

DF, dietary fiber; L-AX, low-dose arabinoxylan; H-AX, high-dose arabinoxylan.

In Experiment 4, the mice were divided into two groups (*n* = 8 per group): DF-free and H-AX. The means and SDs of BW were counterbalanced between groups. As described in Experiment 1, the experimental diets were fed to the mice and the postprandial respiratory metabolic performance of each mouse was measured.

All animal experiments were conducted in the Experimental Animal Facility of Kao Tochigi Institute. The Animal Care Committee of Kao Corporation approved the studies. All experiments strictly followed the guidelines of this committee.

### Blood analysis

Plasma insulin was determined with a mouse insulin enzyme-linked immunosorbent assay (ELISA) kit (Morinaga Institute of Biological Science, Kanagawa, Japan). Plasma total GIP was measured with a rat/mouse GIP (total) ELISA (Millipore, Germany). Plasma glucose was determined by the Glucose C-test kit (Wako Pure Chemical Industries, Osaka, Japan). The incremental area under the curve from 0 to 120 min (iAUC 120 min) was calculated according to the trapezoid rule.

### Statistical analysis

All data are presented as mean ± standard error (SE). Time-dependent changes in the serological results after feeding the diets were compared using a two-way analysis of variance (ANOVA) to evaluate the diet-by-time interaction, the time effect, and the diet effect. When a significant diet-by-time interaction was detected, an intergroup comparison at each time-point during the analytical period was performed with the Bonferroni correction for multiple comparisons after one-way ANOVA. All other statistical tests were performed using the Student’s *t* test (two groups) or the Bonferroni *post hoc* test after one-way ANOVA (over three groups). Dose dependency was evaluated using the Jonckheere trend test. Data analyses were conducted using the Graph Pad Prism program (GraphPad Software, San Diego, CA, USA) and SPSS (IBM Corp., Armonk, NY, USA). Differences at the *p *< 0.05 level were considered statistically significant.

## Results

### Effects of steamed WB on postprandial energy metabolism

**Figure 1.**
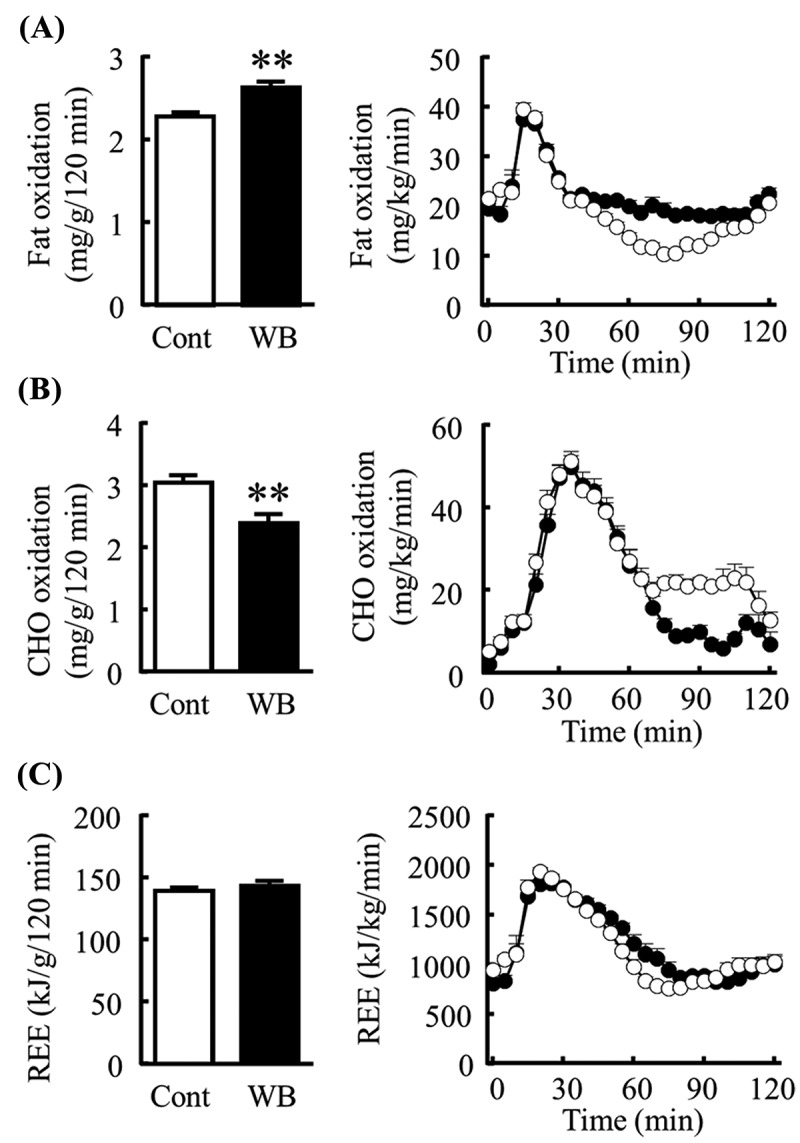
Effects of steamed wheat bran (WB) on postprandial energy metabolism. The graphs show the amount (left) and time-course (right) of (A) fat and (B) carbohydrate (CHO) oxidation, and (C) resting energy expenditure (REE) for 120 min after feeding in mice fed the Control diet (Cont; open circles) or the WB diet (WB; closed circles). All data are presented as mean ± SE (*n* = 8 per group). **p < 0.01 (Student’s *t* test).

In Experiment 1, mice fed the WB diet had a significantly lower respiratory quotient than mice fed the Control diet (data not shown). The WB group had significantly higher fat oxidation and lower carbohydrate oxidation compared with the control group (Figure 1(A, B)). REE (Figure 1(C)) and physical activity (data not shown) were not significantly different between the two groups.

### Effects of steamed WB on postprandial blood variables

**Figure 2.**
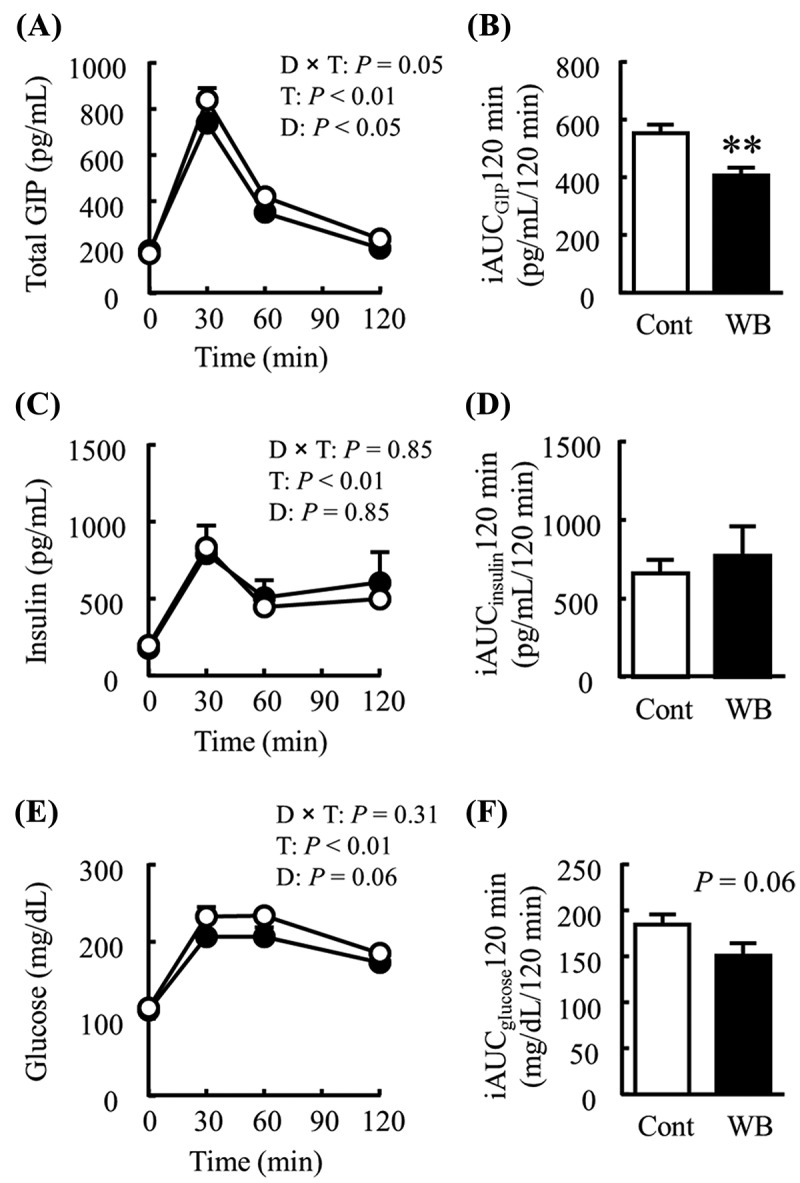
Effects of steamed wheat bran (WB) on postprandial blood variables. The graphs show the plasma concentrations at the indicated times after feeding (A, C, and E) and incremental area under the curve (iAUC; B, D, and F) of (A, B) glucose-dependent insulinotropic polypeptide (GIP), (C, D) insulin, and (E, F) glucose in mice fed the Control diet (Cont; open circles) or the WB diet (WB; closed circles). All data are presented as mean ± SE (*n* = 16 per group). Time-dependent changes were compared using a two-way ANOVA to evaluate the diet-by-time interaction (D × T), the time effect (T), and the diet effect (D). The iAUCs of GIP, insulin, and glucose were analyzed using Student’s unpaired *t* test. **p < 0.01.

In Experiment 2, mice fed the WB diet had significantly lower postprandial blood total GIP levels overall compared with mice fed the Control diet (Diet effect *p* < 0.05), although the difference (diet-by-time interaction) was only marginally significant (*p* = 0.05; Figure 2(A)). There were no significant differences in either the plasma glucose or insulin level between groups (Figure 2(C, E)). The postprandial blood total GIP increase over 120 min (iAUC_GIP_ 120 min) was significantly lower in the WB group than in the control group (Figure 2(B)). Although there was no significant difference in the postprandial blood insulin response (iAUC_insulin_ 120 min) between the two groups (Figure 2(D)), it (iAUC_glucose_ 120 min) tended to be lower (*p =* 0.06) in the WB group than in the control group (Figure 2(F)).

### Effects of arabinoxylan on postprandial blood responses

**Figure 3.**
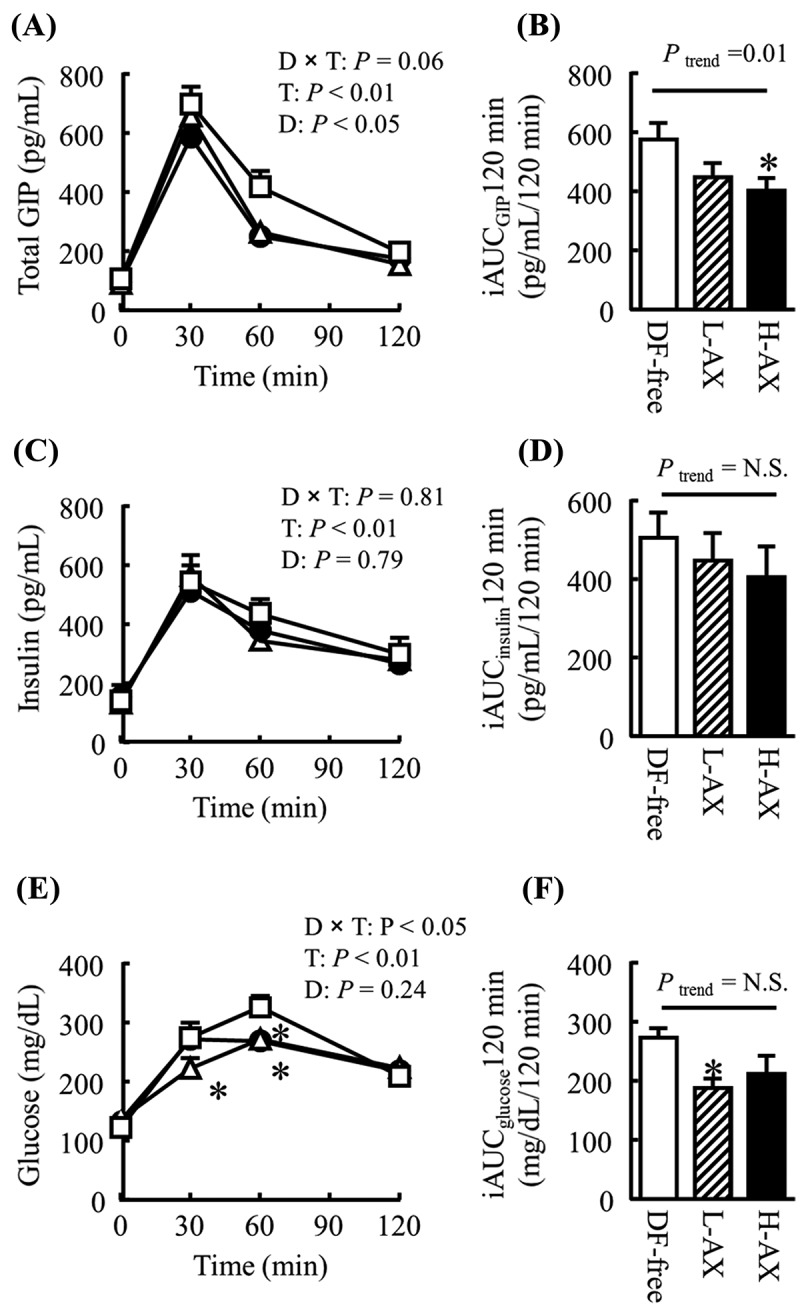
Effects of arabinoxylan on postprandial blood variables. The graphs show the plasma concentrations at the indicated times after feeding (A, C, and E) and the incremental area under the curve (iAUC; B, D, and E) of (A, B) total glucose-dependent insulinotropic polypeptide (GIP), (C, D) insulin, and (E, F) glucose in mice fed the dietary fiber-free diet (DF-free; open squares), the low-dose arabinoxylan diet (L-AX; open triangles), or the high-dose arabinoxylan diet (H-AX; closed circles). All data are presented as mean ± SE (*n* = 8 per group). The incremental areas under the curve (iAUCs) of (B) total GIP, (D) insulin, and (F) glucose of the mice fed each of the diets were evaluated using the Bonferroni *post hoc* test (vs DF-free) after one-way ANOVA. Dose dependency was evaluated using the Jonckheere trend test (*P_trend_*). Time-dependent changes were compared using two-way ANOVA to evaluate the diet-by-time interaction (D × T), the time effect (T), and the diet effect (D). Intergroup comparison at each time-point during the analytical period was conducted with Student’s *t* test. N.S., not significant, *p **< 0.05 vs DF-free group.

In Experiment 3, postprandial blood total GIP, insulin, and glucose levels were not significantly different among the DF-free, cellulose, and lignin diets (Supplemental Figure 1). The postprandial blood total GIP response was significantly lower in mice fed the arabinoxylan diet compared with the DF-free group (Figure 3(A)) (*p* = 0.09 in the L-AX and *p* = 0.03 in the H-AX, by two-way ANOVA followed by the Bonferroni *post hoc* test). Supplementation with arabinoxylan significantly decreased the postprandial blood total GIP response (iAUC_GIP_ 120 min) in a dose-dependent manner (Figure 3(B)) (*P_trend_* = 0.01, by the Jonckheere trend test). Postprandial blood insulin levels and response over 120 min (iAUC_insulin_ 120 min) were not significantly different among the groups (Figure 3(C, D)). The postprandial blood glucose level was significantly lower in the L-AX group after 30 and 60 min, and in the H-AX group after 60 min compared with the DF-free group (Figure 3(E)). The overall postprandial blood glucose response (iAUC_glucose_120 min) was lower after being fed the arabinoxylan than after being fed the DF-free diet (Figure 3(F)).

### Effects of arabinoxylan on postprandial energy metabolism

**Figure 4.**
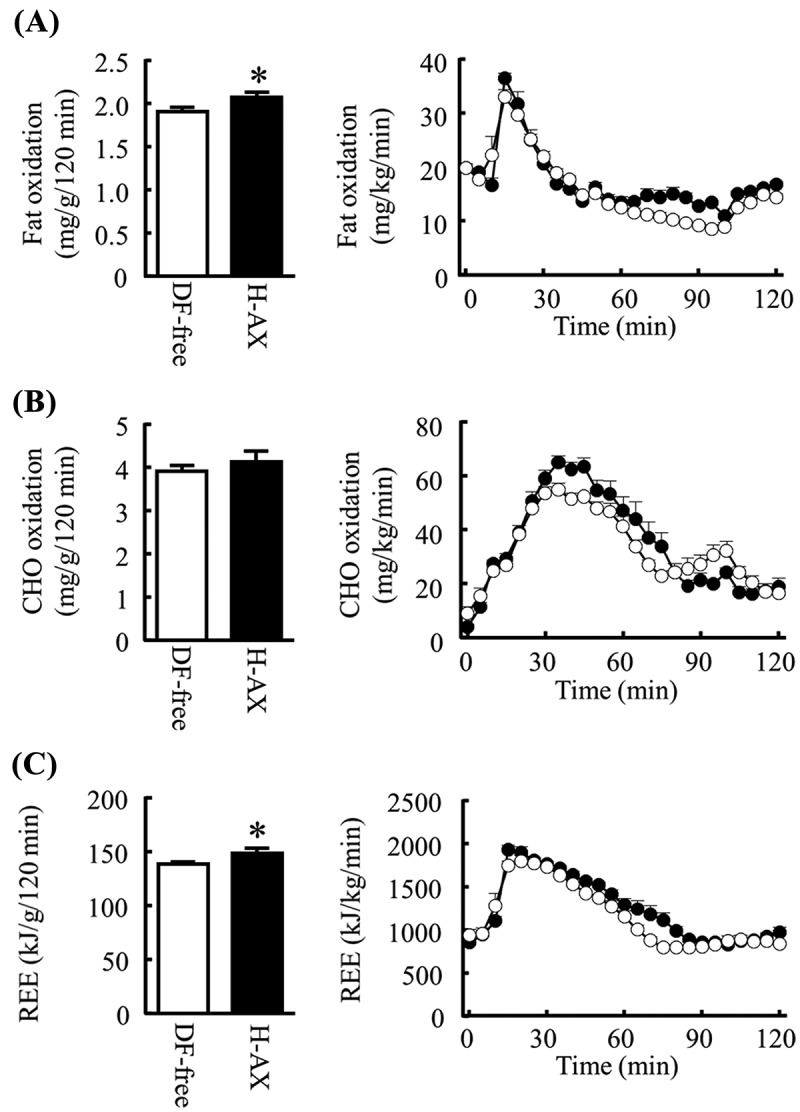
Effects of arabinoxylan on postprandial energy metabolism. The graphs show the amount (left) and time-course (right) of (A) fat and (B) carbohydrate (CHO) oxidation, and (C) resting energy expenditure (REE) for 120 min after feeding in mice fed the dietary fiber-free diet (DF-free; open circles) or the high-dose arabinoxylan diet (H-AX; closed circles). All data are presented as mean ± SE (*n* = 8 per group). *p **< 0.05 (Student’s *t* test). Effects of steamed wheat bran (WB) on postprandial plasma dipeptidyl peptidase-4 (DPP-4) activity.

In Experiment 4, postprandial fat oxidation for 120 min was significantly greater in the H-AX group than in the DF-free group (Figure 4(A)). Carbohydrate oxidation was not significantly different between the groups (Figure 4(B)). Postprandial REE was also significantly greater after feeding on the H-AX diet compared with the DF-free diet (Figure 4(C)). Physical activity was not significantly different between the groups (data not shown).

## Discussion

The findings of the present study demonstrated that a single ingestion of dietary steamed WB reduced the postprandial blood GIP response and increased postprandial fat oxidation in mice. Furthermore, we revealed that the arabinoxylan fraction prepared from the steamed WB was responsible for the reduction in the postprandial blood GIP response and the increase in postprandial fat utilization after consuming the WB diet.

Several mechanisms are suggested to underlie the anti-obesity effects of chronic ingestion of WB. Southgate et al. [14] reported that dietary bran reduces nutrient absorption. Seminal studies performed by Han et al. [11] demonstrated that WB increases the expression of proteins related to fatty acid oxidation by modulating the sterol-regulatory element binding protein pathway, and increases lipolysis and browning of adipocytes [12] in mice. On the other hand, many studies have demonstrated that chronic disruption and suppression of GIP signaling, either genetically, or by vaccination against GIP, or by administration of materials that reduce postprandial blood GIP, leads to high fat utilization and accelerates lipolysis in adipose tissue [21–25,27–29,34]. The findings of the present study are consistent with previous findings, and add new insight into the anti-obesity mechanisms of WB.

In this study, postprandial energy substrate oxidation shifted from carbohydrate to fat after mice consumed the WB diet, without a significant difference in postprandial energy expenditure. Increased fat oxidation with similar energy expenditure is associated with a decrease in BW and fat mass [35–37]. In addition, postprandial fat oxidation is negatively associated with the body fat ratio, and lower postprandial fat oxidation is an early predictor of BW gain [38,39]. These findings suggest that fat utilization is a major determinant of obesity. Therefore, enhanced postprandial fat utilization after a single ingestion of steamed WB contributes towards improving obesity.

Daousi et al. [26] reported that intravenous infusion of GIP reduces REE in healthy humans, suggesting that GIP has acute effects on energy catabolism. In the present study, blood GIP levels were reduced after consumption of steamed WB and, simultaneously with the decrease in the blood GIP levels, fat utilization was increased in mice fed the WB diet. Accordingly, lower blood GIP levels after steamed WB consumption probably contribute to the increase in postprandial fat oxidation. GIPRs are expressed not only in the pancreas but also in various metabolic organs [40]. Although the mechanisms underlying the anti-obesity effects of WB remains uncertain, the extrapancreatic GIP/GIPR signaling activity may play a significant role in increasing fat oxidation. On the other hand, active GIP is degraded by dipeptidyl peptidase-4 to form an NH_2_-terminally truncated metabolite, GIP(3-42) [41,42]. Although the function of GIP(3-42) is controversial, it has been suggested that it could be an antagonist for GIPRs [43–45]. Studies to determine the blood active GIP and GIP(3-42) levels are required to clarify the precise effects on GIP/GIPR signaling activity after ingestion of steamed WB and arabinoxylan. Increased blood glucose and insulin levels also reduce fat oxidation [46–48]. While the postprandial blood insulin response was similar, the blood glucose response tended to be lower after WB feeding in the present study. WB or an arabinoxylan-rich diet may slow the rate of gastric emptying, which results in delayed glucose absorption [15–17,49]. Delayed glucose absorption leads to reduced GIP secretion from K cells and lower blood glucose levels, which may partly explain the increase in postprandial fat oxidation. Further studies are needed to clarify the mechanism of the increased postprandial fat oxidation after ingestion of steamed WB.

In the present study, the postprandial blood insulin response was similar despite the fact that the postprandial blood GIP response was significantly decreased after WB consumption. This finding implies the existence of another non-GIP mechanism regulating postprandial insulin secretion after consumption of the steamed WB and arabinoxylan. Glucagon-like peptide-type 1 (GLP-1) and xenin also stimulate insulin secretion [50,51]. Postprandial levels of xenin, which is co-released from K cells with GIP and amplifies GIP-mediated insulin secretion, did not differ after WB consumption (data not shown). Hartvigsen et al. [52] demonstrated that a single ingestion of bread containing wheat arabinoxylan stimulates the secretion of GLP-1 and insulin compared with refined wheat bread in subjects with metabolic syndrome, suggesting that GLP-1 secretion could be induced by ingesting a meal containing steamed WB. In addition, other gastrointestinal hormones, such as ghrelin and peptide YY, affect the postprandial blood response and energy metabolism [53,54]. A limitation of the present study is that the amounts of plasma samples were insufficient to measure other gastrointestinal hormones. The effect of steamed WB and arabinoxylan on the postprandial blood levels of other gastrointestinal hormones is unclear, but warrants future examination.

Arabinoxylan is a hemicellulose consisting of a backbone of β-(1,4)-linked xylose residues, which are substituted with arabinose residues at the C(O)-2 and/or C(O)-3 position [55]. Arabinoxylan has various beneficial effects for health; for example, decreased obesity [56,57], increased glucose metabolism [49], and immune-stimulating effects [58]. While the arabinoxylan fraction prepared from steamed WB in this study contained 19.4% phytic acid, the postprandial blood GIP response was not altered after consumption of other WB fractions that contained 18.8% phytic acid as the major component with 4.3% arabinoxylan (Supplemental Figure 2). Accordingly, our results indicate that arabinoxylan is a major active component for decreasing the postprandial blood GIP response and increasing postprandial fat utilization after consumption of WB. Not decreasing carbohydrate oxidation may contribute to the increase in postprandial energy expenditure after arabinoxylan consumption. The findings suggest that inhibitory components of postprandial carbohydrate utilization are removed during preparation of the arabinoxylan fraction from steamed WB. An increase in the arabinoxylan content and a decrease in the inhibitory components in the WB may result in greater postprandial energy expenditure and thereby improve the anti-obesity effects of steamed WB.

Although not detracting from the importance of the current findings, the relationship between the lower blood GIP response and higher fat utilization after WB consumption remains unclear because blood GIP and metabolic responses were measured in separate groups of mice, which is another limitation of the present study. Our previous study provided evidence that the postprandial blood GIP response is negatively associated with postprandial fat utilization and REE after RS4-type resistant starch consumption in healthy humans [59]. Studies to elucidate the association of the lower blood GIP response with higher fat utilization after WB consumption in humans are in progress.

In conclusion, we clarified that a single ingestion of steamed WB reduced postprandial GIP secretion and increased postprandial fat oxidation in mice. We also provided evidence that arabinoxylan, a major indigestible carbohydrate component of steamed WB, was responsible for the beneficial postprandial effects of the steamed WB. These physiological effects provide new insight into the anti-obesity mechanisms of steamed WB. These findings also imply that increasing the arabinoxylan content or removing the inhibitory components relevant to decreased postprandial carbohydrate oxidation could lead to the development of refined WB for the amelioration of obesity.

## Supplementary Material

ZFNR_A_1361778_Supplemental_Material.zip
