# Supplementary material for: Dietary steamed wheat bran increases postprandial fat oxidation in association with a reduced blood glucose-dependent insulinotropic polypeptide response in mice
Source: Food Nutr Res. 2017 Aug 23;61(1):1361778. doi: 10.1080/16546628.2017.1361778 (PMC5614337; doi:10.1080/16546628.2017.1361778)

**Supplemental data**

Materials and methods

In supplemental experiment 1, overnight-fasted mice were divided into three groups (*n* = 8/group): DF-free, cellulose, and lignin groups. The mean and standard deviation of the body weight and fasting blood glucose concentration were counterbalanced among groups. Mice in each group were individually fed a DF-free diet, a diet containing cellulose, or a diet containing lignin (Supplemental Table 1), with each diet containing the same quantity of dietary fiber as in a 3.83-kJ WB diet (Table 3). The dietary fiber content of steamed WB was based on reference 7 (i.e., 12.1% cellulose and 4.9% lignin). As described in Experiment 2, the experimental diets were fed to the mice and postprandial blood samples were collected.

The reduced arabinoxylan fraction (R-AX fraction) was prepared according to previously reported methods (J of Food Composition and Analysis. 2011; 24: 1057-63). Briefly, steamed wheat bran was washed with hexane, ethanol, and distilled water. The resulting residue was extracted with 1.0 M HCl aq. The extract was neutralized to pH 8.0 with NaOH aq. to form the precipitate (precipitate A). Precipitate A was collected after centrifugation, and the supernatant was further precipitated by adding an equivalent volume of ethanol. The resulting precipitate (precipitate B) was collected after centrifugation. The mixture of precipitates A and B (R-AX fraction) was analyzed for its components and examined as described in the main text. The amount of arabinoxylan and phytic acid of R-AX fraction was determined as described in the main text. The protein content was measured using a Protein Assay Rapid Kit Wako (Wako Pure Chemical Industries, Osaka, Japan). In supplemental experiment 2, overnight-fasted mice were divided into two groups (*n* = 8/group): DF-free and R-AX groups. The mean and standard deviation of the body weight and fasting blood glucose concentration were counterbalanced among groups. Mice in each group were individually fed a DF-free diet or a diet containing R-AX fraction (Supplemental Table 1). As described in Experiment 2, the experimental diets were fed to the mice and postprandial blood samples were collected.

**Supplemental TABLE 1.** Nutrient content of the diets.

| Ingredients (mg/g diet) | DF-free diet | Cellulose-diet | Lignin-diet | R-AX diet |
| --- | --- | --- | --- | --- |
| Gelatinized potato starch | 510 | 489 | 502 | 473 |
| Sucrose | 130 | 125 | 128 | 121 |
| Corn oil | 97 | 93 | 95 | 90 |
| Milk casein | 218 | 209 | 214 | 202 |
| AIN-76 mineral mixture | 35 | 33 | 34 | 32 |
| AIN-76 vitamin mixture | 10 | 10 | 10 | 9 |
| Cellulose | 0 | 41 | 0 | N.D. |
| Lignin  R-AX fraction | 0  0 | 0  0 | 17  0 | N.D.  73 |
| Arabinoxylan | 0 | 0 | 0 | 3.1 |
| Total energy (kJ/g) | 18.0 | 17.2 | 17.6 | 16.7 |
| Fat (% energy) | 20.3 | 20.3 | 20.3 | 20.3 |
| Protein (% energy) | 20.3 | 20.3 | 20.3 | 20.3 |
| Carbohydrates (% energy) | 59.4 | 59.4 | 59.4 | 59.4 |

**Supplement TABLE 2.** Composition of the R-AX fraction.

| Components (%) | fraction |
| --- | --- |
| Arabinose/Xylose | 4.3 |
| Phytic acid | 18.8 |
| Protein | 0.7 |
| Others | 76.2 |

**Supplemental Figure 1.** Effects of cellulose and lignin on postprandial blood variables.

  Plasma concentrations at the indicated times after the feeding (A, C, and E) and incremental area under the curve (iAUCs; B, D, and F) of (A, B) total GIP, (C, D) insulin, and (E, F) glucose in mice fed the DF-free diet (DF-free; open circles), diet containing cellulose (Cell; empty squares), or diet containing lignin (Lig; filled triangles). All data are presented as the mean ± standard error (SE). Time-dependent changes were compared using a two-way ANOVA to evaluate the diet-by-time interaction (D × T), the time effect (T), and the diet effect (D). The iAUCs of total GIP, insulin, and glucose were evaluated using the Bonferroni post hoc test (vs. DF-free) after one-way ANOVA.

**
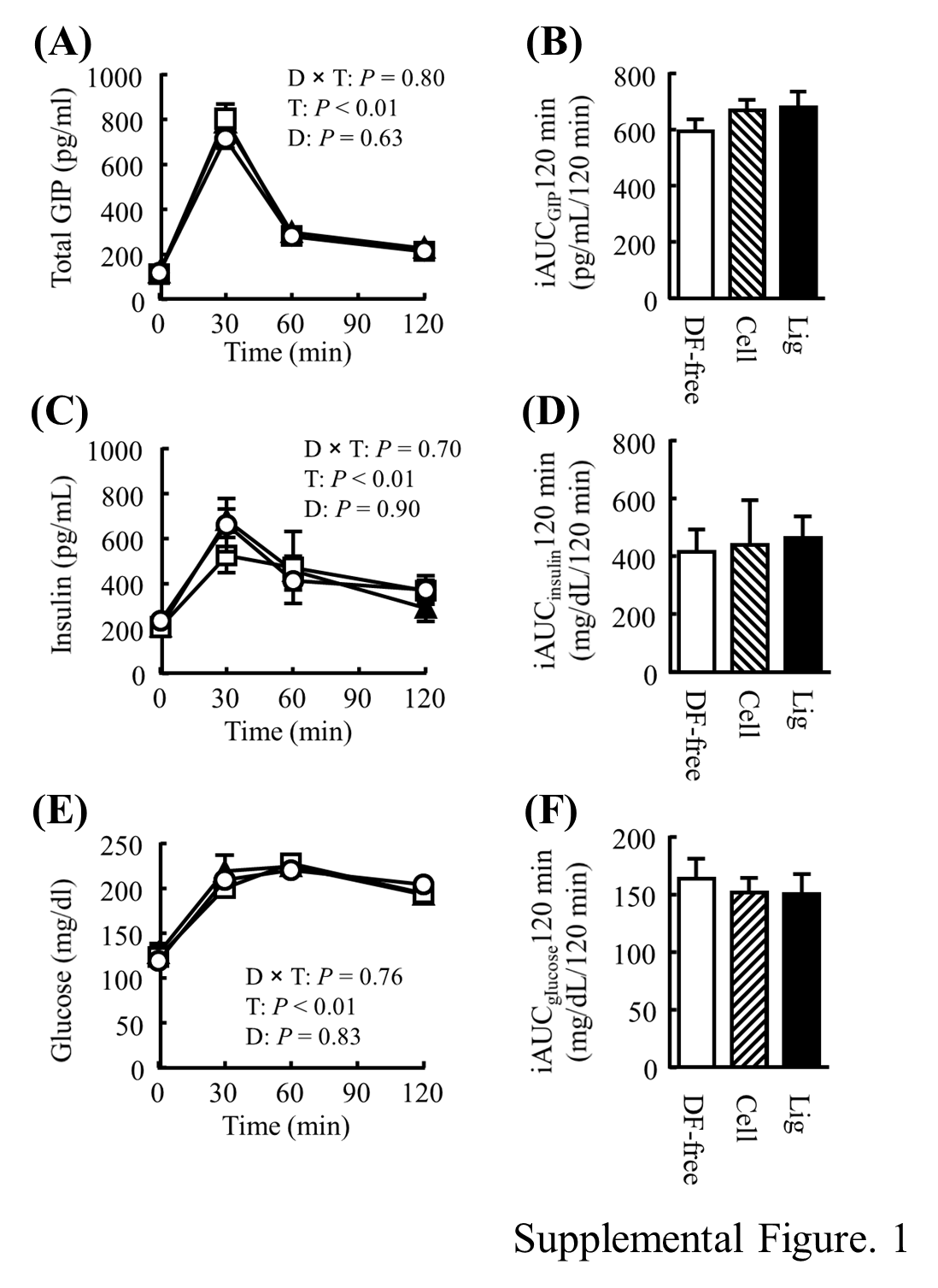
**

**Supplemental Figure 2.** Effects of the R-AX fraction on postprandial blood variables.

  Plasma concentrations at the indicated times after the feeding (A, C, and E) and incremental area under the curve (iAUCs; B, D, and F) of (A, B) total GIP, (C, D) insulin, and (E, F) glucose in mice fed the DF-free diet (DF-free; open circles) or diet containing the R-AX fraction (R-AX; filled circles). All data are presented as the mean ± standard error (SE). Time-dependent changes were compared using a two-way ANOVA to evaluate the diet-by-time interaction (D × T), the time effect (T), and the diet effect (D). The iAUCs of total GIP, insulin, and glucose were evaluated using a t-test.


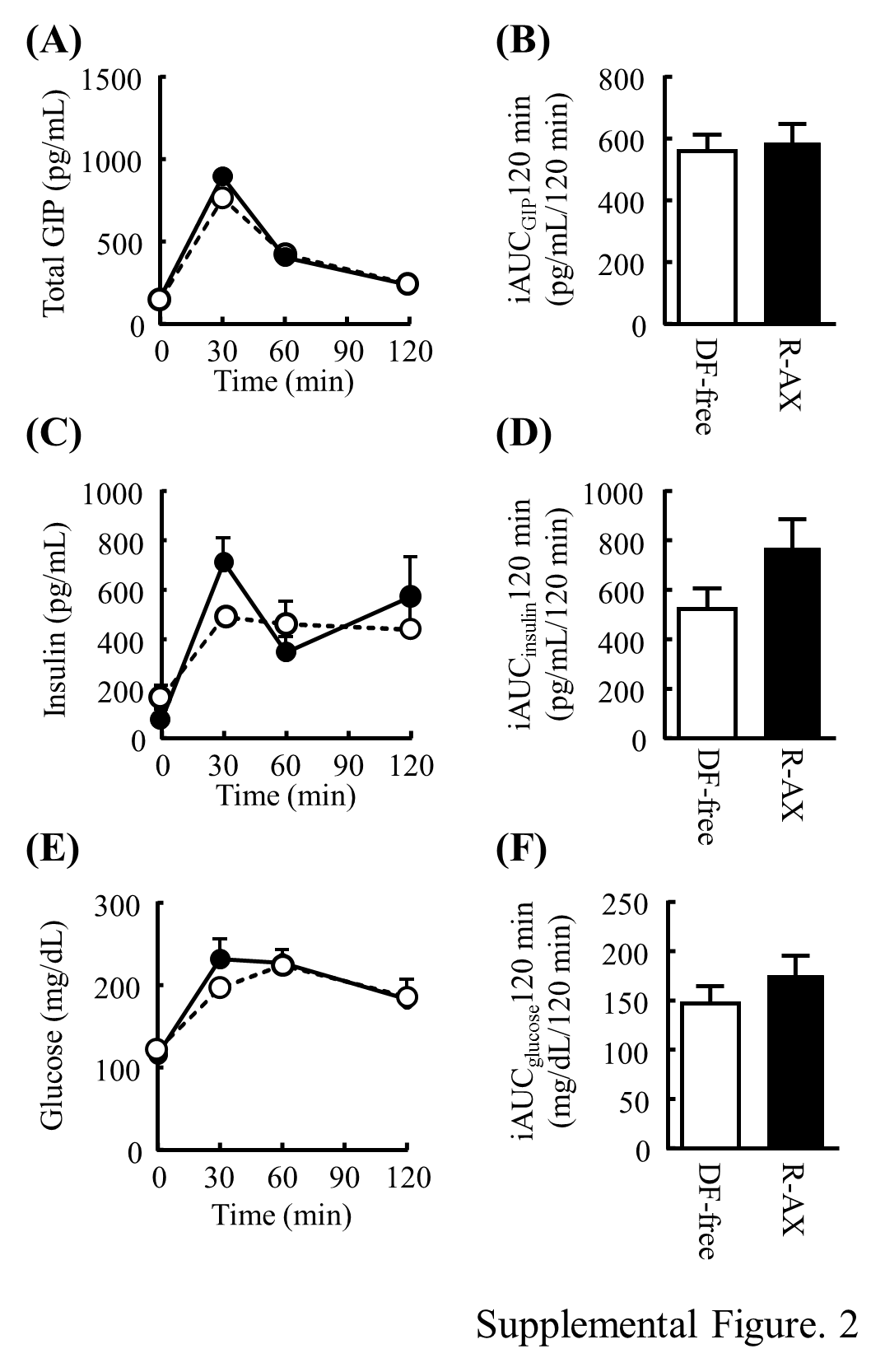

Supplement: ZFNR_A_1361778_Supplemental_Material.zip [file ZFNR_A_1361778_SM9128.zip › Supplemental Material.docx]
