# Supplementary figures and images for: Dietary steamed wheat bran increases postprandial fat oxidation in association with a reduced blood glucose-dependent insulinotropic polypeptide response in mice
Source: Food Nutr Res. 2017 Aug 23;61(1):1361778. doi: 10.1080/16546628.2017.1361778 (PMC5614337; doi:10.1080/16546628.2017.1361778)

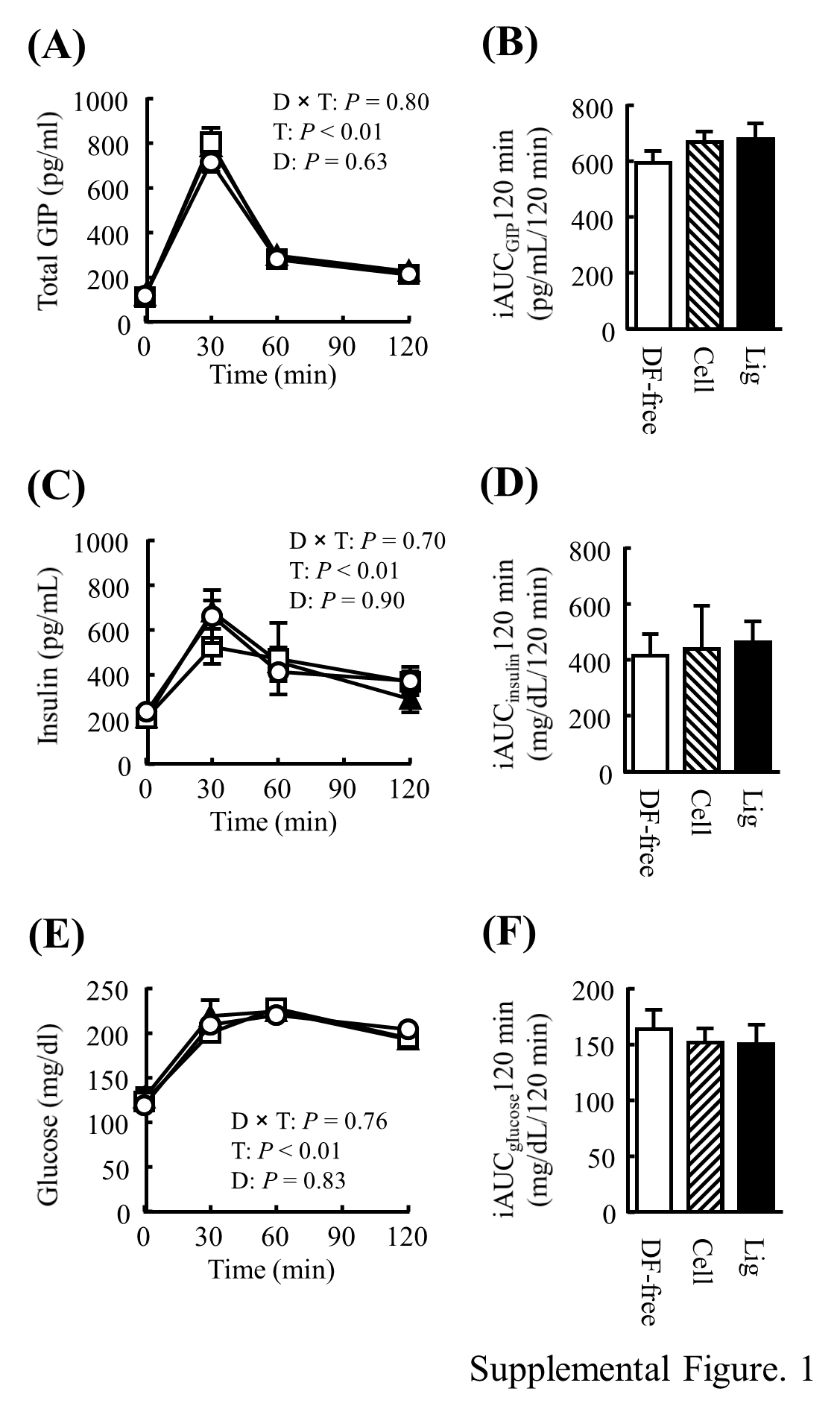

Supplement: ZFNR_A_1361778_Supplemental_Material.zip [file ZFNR_A_1361778_SM9128.zip › supplemental figure 1.tif]

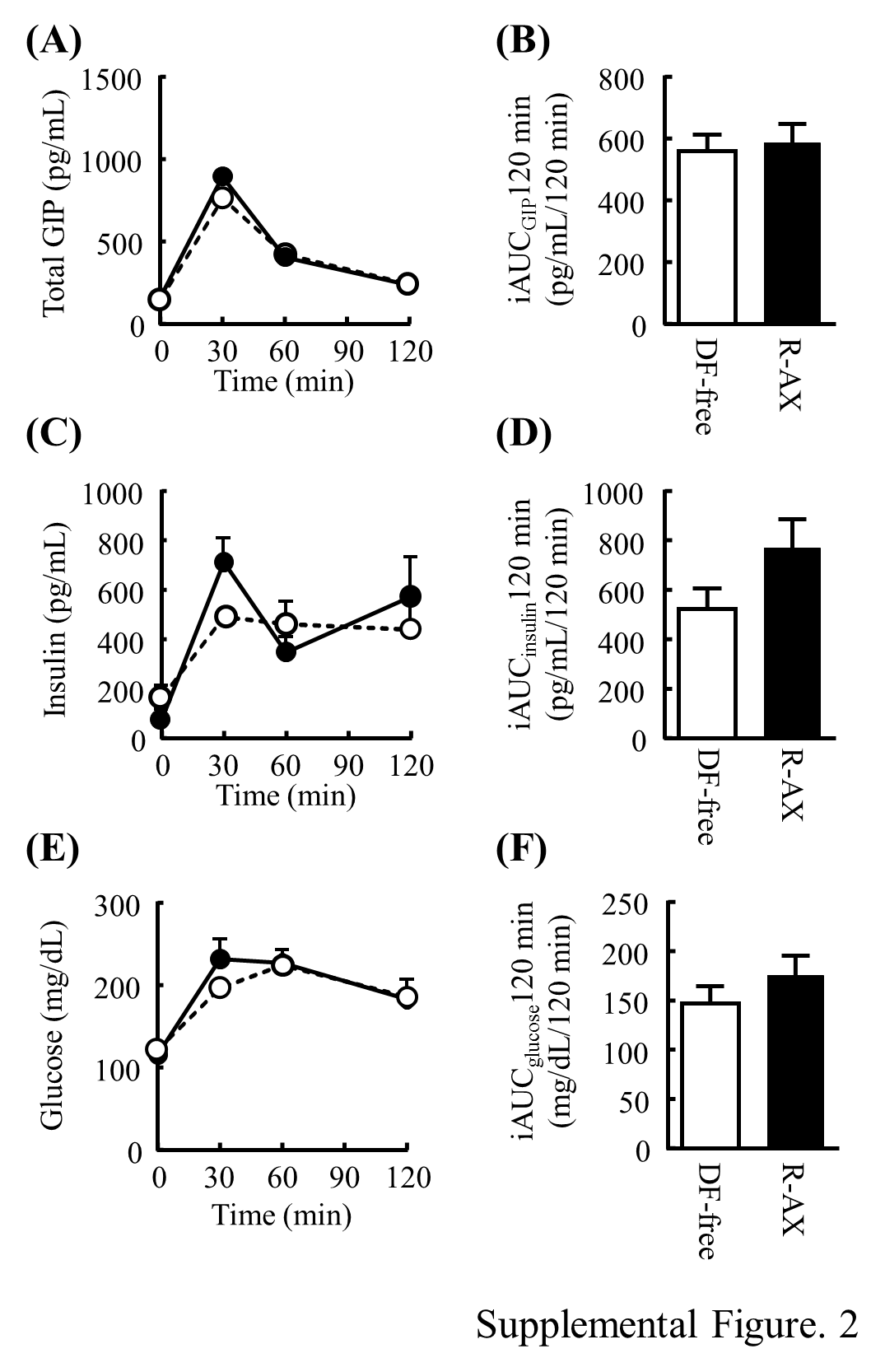

Supplement: ZFNR_A_1361778_Supplemental_Material.zip [file ZFNR_A_1361778_SM9128.zip › supplemental figure 2.tif]
